# Supplementary material for: The DBD-α4 helix of EWS::FLI is required for GGAA microsatellite binding that underlies genome regulation in Ewing sarcoma
Source: bioRxiv. 2024 Jan 31:2024.01.31.578127. Preprint. [Version 1] doi: 10.1101/2024.01.31.578127 (PMC10862889; doi:10.1101/2024.01.31.578127)
Supplement: Supplement 1 [file NIHPP2024.01.31.578127v1-supplement-1.pdf]

bioRxiv preprint doi: https://doi.org/10.1101/2024.01.31.578127; this version posted January 31, 2024. The copyright holder for this preprint (which was not certified by peer review) is the author/funder, who has granted bioRxiv a license to display the preprint in perpetuity. It is made available under aCC-BY 4.0 International license.

562 ## Supplementary Data

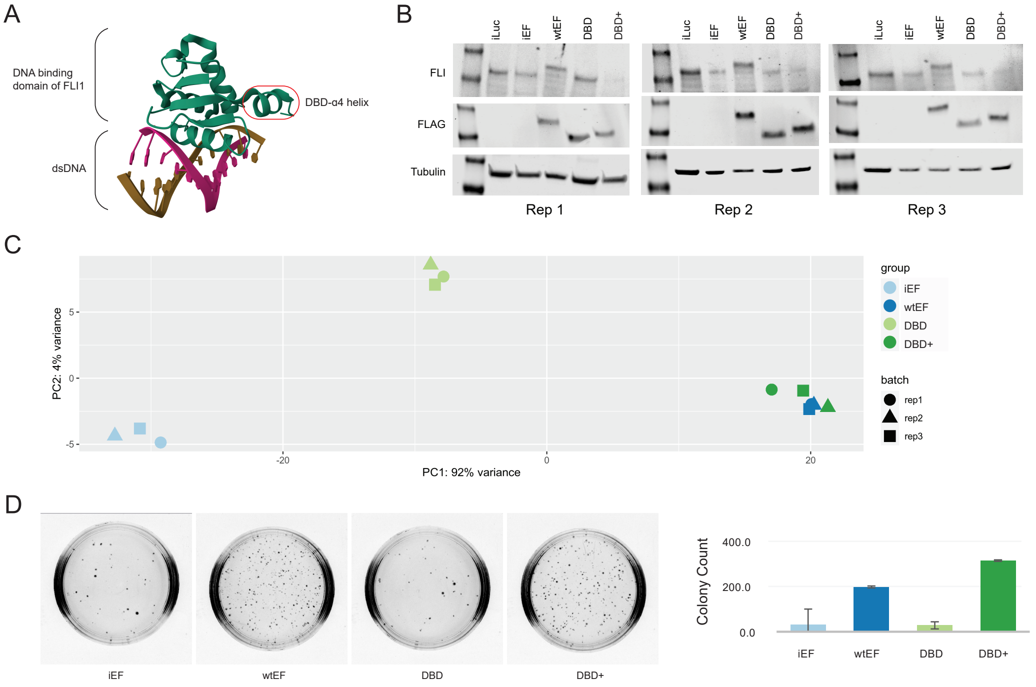

Supplementary Figure 1: A. DBD-α4 helix of FLI1 depicted on dsDNA (PDB) B. Knock-down of endogenous EWS::FLI detected with FLI1 ab and rescue of wtEF, DBD, and DBD+ detected with FLAG ab. C. A PCA plot of RNA-seq experiments in A673 cells. D. Representative image of soft agar colony plates and quantification of three biological replicates.

bioRxiv preprint doi: https://doi.org/10.1101/2024.01.31.578127; this version posted January 31, 2024. The copyright holder for this preprint (which was not certified by peer review) is the author/funder, who has granted bioRxiv a license to display the preprint in perpetuity. It is made available under aCC-BY 4.0 International license.

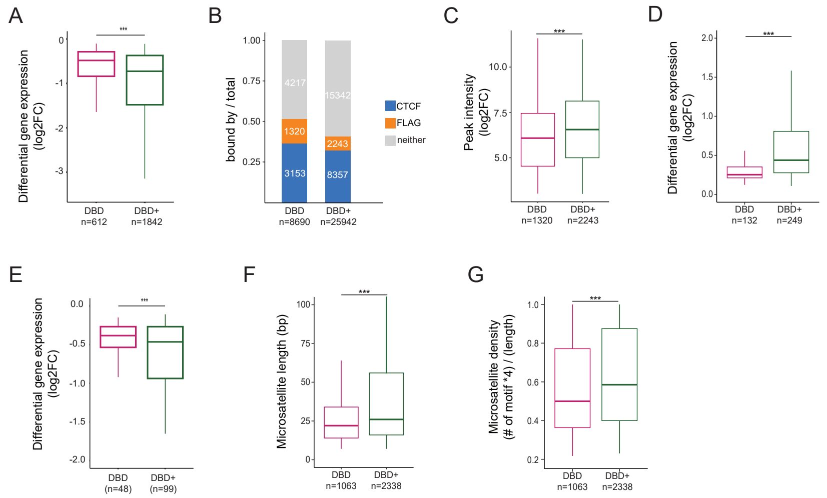

Supplementary Figure 2. A. Expression level of significant genes overlapped with unique TADs in DBD (mean=-0.64) and DBD+ (mean=-1.16) bound by FLAG at GGAA microsatellites. B. Proportion of TAD boundaries bound by FLAG, CTCF or neither. C-G. Comparison of DBD and DBD+ unique TAD boundaries. C. Binding intensity of unique FLAG peaks (FDR < 0.05, FC > 8, counts > 80, IDR < 0.01) at boundaries of DBD and DBD+ unique TADs. D. Expression level of significantly upregulated genes overlapped with boundaries of unique TADs in DBD and DBD+ bound by FLAG at GGAA microsatellites. E. Expression level of significantly downregulated genes overlapped with boundaries of unique TADs in DBD and DBD+ bound by FLAG at GGAA microsatellites. F. Length of microsatellites bound by unique FLAG peaks at the boundaries of DBD and DBD+ conditions in bp. G. Percent of GGAA motif in the microsatellites calculated as (of motif x 4)/(length of microsatellites) at the boundaries of DBD and DBD+ unique TADs bound by unique FLAG peaks. Boxplots depict the minimum, first quartile, median, third quartile, and maximum. *** P value < 0.001

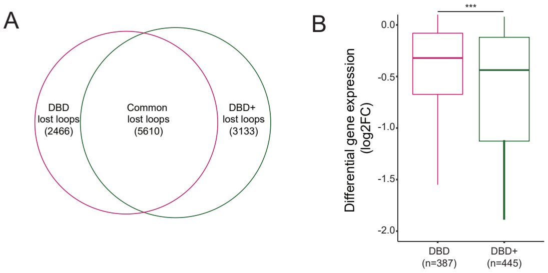

Supplementary Figure 3. A. Venn diagram of overlap between DBD and DBD+ uniquely lost loops (compared to KD). B. Expression level of downregulated genes overlapped with uniquely gained loop anchors of DBD and DBD+. Means = -0.68, -1.25, 0.35, -1.08 * P value < 0.05, *** P value < 0.001. Boxplots depict the minimum, first quartile, median, third quartile, and maximum.

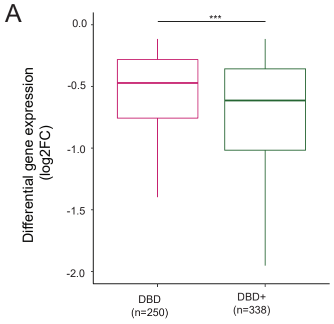

Supplementary Figure 4. A. Expression level of downregulated genes at DBD and DBD+ super enhancers. *** P value < 0.001 Boxplots depict the minimum, first quartile, median, third quartile, and maximum.

bioRxiv preprint doi: https://doi.org/10.1101/2024.01.31.578127; this version posted January 31, 2024. The copyright holder for this preprint (which was not certified by peer review) is the author/funder, who has granted bioRxiv a license to display the preprint in perpetuity. It is made available under aCC-BY 4.0 International license.

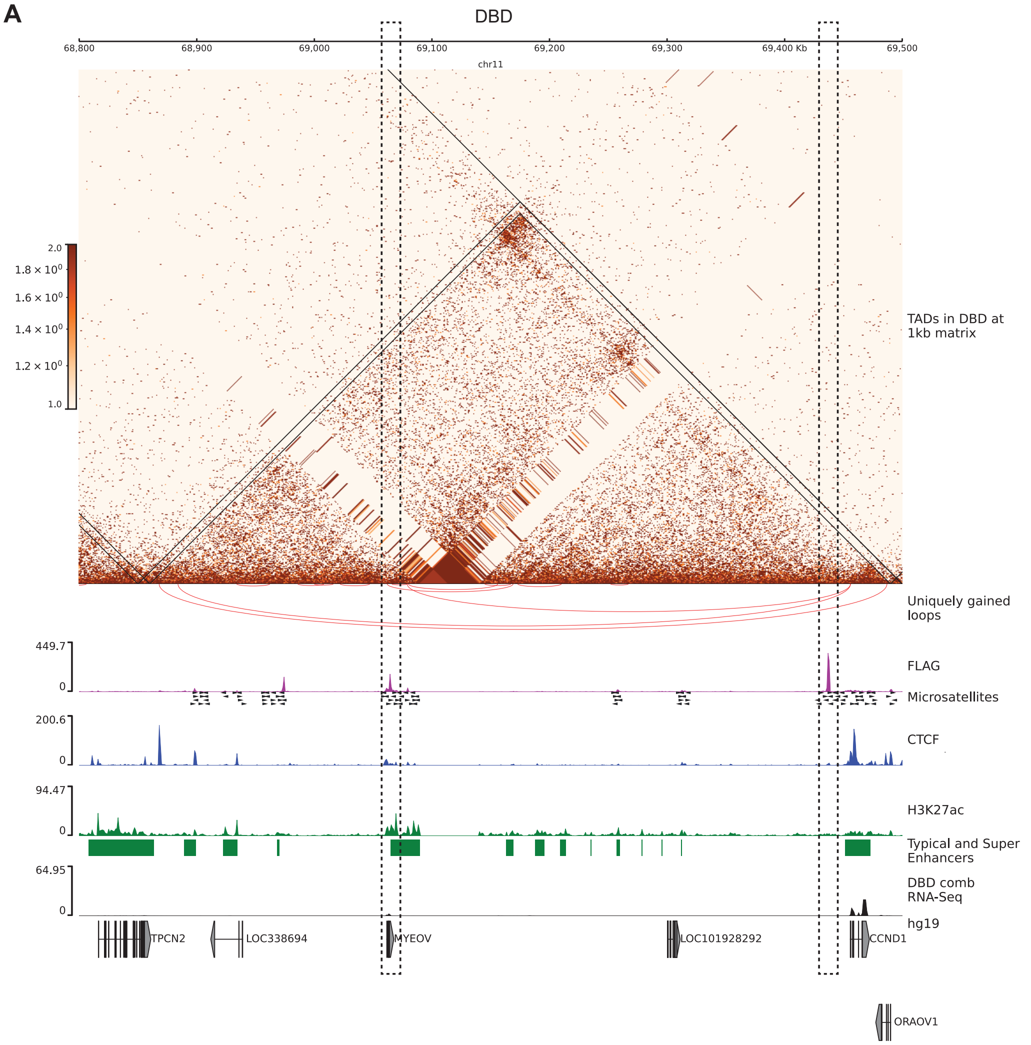

Supplementary Figure 5 . A. CCND1 hub in 700kb region on chr 11 in DBD cells. TADs are depicted on 1kb matrices (DBD/KD). Uniquely gained loops are shown as red inverted arcs. FLAG CUT&Tag bigwig tracks depicted in magenta. GGAA microsatellites in hg19. CTCF CUT&Tag track is in blue middle row. H3K27ac tracks are in green. Enhancers and super-enhancers are shown as green bars. Gene expression is in black tracks.

bioRxiv preprint doi: https://doi.org/10.1101/2024.01.31.578127; this version posted January 31, 2024. The copyright holder for this preprint (which was not certified by peer review) is the author/funder, who has granted bioRxiv a license to display the preprint in perpetuity. It is made available under aCC-BY 4.0 International license.

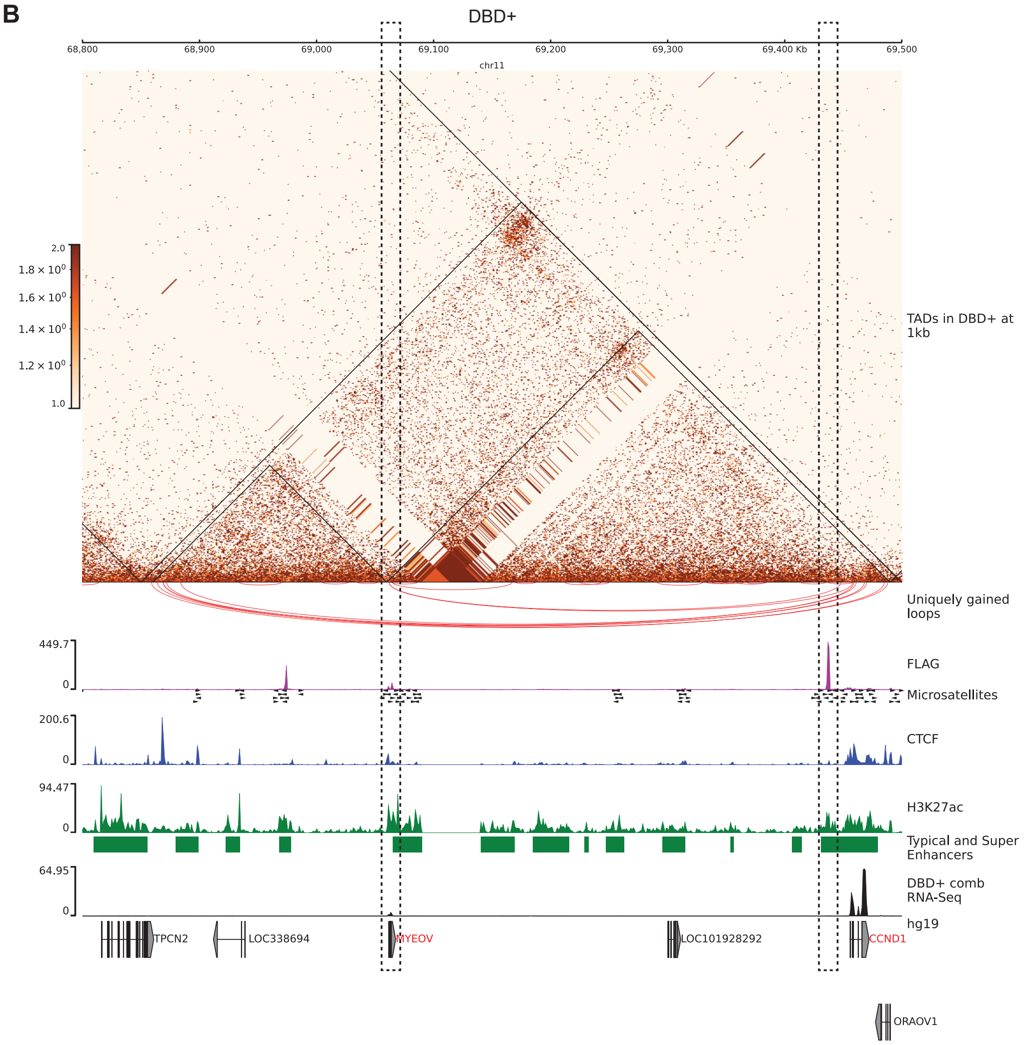

Supplementary Figure 5. B. CCND1 hub in 700kb region on chr 11 in DBD+ cells. TADs are depicted on 1kb matrices (DBD+/KD). Uniquely gained loops are shown as red inverted arcs. FLAG CUT&Tag bigwig tracks depicted in magenta. GGAA microsatellites in hg19. CTCF CUT&Tag track is in blue middle row. H3K27ac tracks are in green. Enhancers and super-enhancers are shown as green bars. Gene expression is in black tracks.

bioRxiv preprint doi: https://doi.org/10.1101/2024.01.31.578127; this version posted January 31, 2024. The copyright holder for this preprint (which was not certified by peer review) is the author/funder, who has granted bioRxiv a license to display the preprint in perpetuity. It is made available under aCC-BY 4.0 International license.

Table 1. Supplementary Table 1. Differential expression of FCGRT hub genes in DBD and DBD+ compared to KD.

Gene Symbol	DBD FC	DBD padj	DBD+ FC	DBD+ padj
ALDH16A1	1.207	0.04635288	1.107	0.329212288
RPL13A	-1.132	0.021442589	-1.209	7.46E-05
RPL13AP5	-1.109	0.490879542	-1.149	0.290788743
RPS11	-1.068	0.337413798	-1.177	0.001887497
FCGRT	1.639	0.0002149	2.44	2.54E-13
RCN3	-1.588	6.85E-06	-1.406	0.000957843
NOSIP	1.065	0.446781941	-1.041	0.636826207
PRRG2	1.117	0.759840545	-1.042	0.916808784
PRR12	1.177	0.006810095	1.088	0.20377503
RRAS	-1.655	1.63E-17	-1.383	5.10E-08
SCAF1	1.065	0.357378849	-1.07	0.283421905
IRF3	1.081	0.4016866	1.196	0.011473037
BCL2L12	1.255	0.006880506	1.359	6.60E-05
PRMT1	1.151	0.015484657	1.145	0.014515775
ADM5	1.122	0.740397828	1.064	0.863762064
